# Progression of frailty and prevalence of osteoporosis in a community cohort of older women—a 10-year longitudinal study

**DOI:** 10.1007/s00198-018-4593-7

**Published:** 2018-06-12

**Authors:** P. Bartosch, F. E. McGuigan, K. E. Akesson

**Affiliations:** 1 Lund University, Department of Clinical Sciences Malmö, Clinical and Molecular Osteoporosis Research Unit, 20502 Malmö, Sweden; 2 Department of Orthopaedics, Skåne University Hospital, 205 02 Malmö, Sweden

**Keywords:** Bone density, Community-dwelling, Frailty, Mortality, Women

## Abstract

**Summary:**

In community dwelling, 75-year-old women followed 10 years, a frailty index was created at each of three visits. Frailty score increased by ~ 6–7% annually. A higher frailty score was equivalent to being 5–10 years chronologically older. Frailty was associated with low bone density and higher risk of dying.

**Introduction:**

To understand the distribution of frailty among a population-based sample of older community-dwelling women, progression over 10 years, and association with mortality and osteoporosis.

**Methods:**

The study is performed in a cohort designed to investigate osteoporosis. The OPRA cohort consists of 75-year-old women, *n* = 1044 at baseline, and follow-up at age 80 and 85. A frailty index (scored from 0.0–1.0) based on deficits in health across multiple domains was created at all time-points; outcomes were mortality up to 15 years and femoral neck bone density.

**Results:**

At baseline, the proportion least frail, i.e., most robust (FI 0.0–0.1) constituted 48%, dropping to 25 and 14% at age 80 and 85. On average, over 10 years, the annual linear frailty score progression was approximately 6–7%. Among the least frail, 11% remained robust over 10 years. A higher frailty score was equivalent to being 5 to 10 years older. Mortality was substantially higher in the highest quartile compared to the lowest based on baseline frailty score; after 10 years, 48.7% had died vs 17.2% (*p* = 1.7 × 10^−14^). Mortality risk over the first 5 years was highest in the frailest (Q4 vs Q1; HR_unadj_ 3.26 [1.86–5.73]; *p* < 0.001) and continued to be elevated at 10 years (HR_unadj_ 3.58 [2.55–5.03]; *p* < 0.001). Frailty was associated with BMD after adjusting for BMI (overall *p* = 0.006; Q1 vs Q4 *p* = 0.003).

**Conclusions:**

The frailty index was highly predictive of mortality showing a threefold increased risk of death in the frailest both in a shorter and longer perspective. Only one in ten older women escaped progression after 10 years. Frailty and osteoporosis were associated.

## Introduction

The expected demographic change towards an increasingly elderly population [1] indicates the importance of understanding frailty and the clinical implications of frailty for successful aging. Frailty has become central in geriatric medicine, contributing as it does to a higher risk for many adverse health outcomes [2] and institutionalization [3]. Frailty encompasses the functional decline in multiple physiological systems, among others, neurodegeneration, sarcopenia, and cognitive changes [4–6]. However, perhaps the most dramatic declines, in terms of function and structure, are in the musculoskeletal system, affecting balance, mobility, disability, and ultimately the ability to live independently. In the field of osteoporosis, research into frailty is still not a major focus, despite being potentially highly relevant since the most severe fractures occur in the old, hip fractures in particular. The few studies available suggest an association with osteoporosis outcomes [7–12].

Frailty as a concept has been most extensively studied in order to understand factors associated with rapid decline in health status ultimately leading to death, and in addition identify targets for intervention [13, 14]. However, a gap in knowledge still exists since comparatively few frailty studies are designed to provide long-term data on older community-dwelling women [15], especially its pattern of progression. Furthermore, despite the prevalence of osteoporosis and its consequences in older populations, cohorts designed to specifically address osteoporosis may not have sufficient data to adequately capture frailty. Likewise, cohorts designed to address frailty or other conditions may lack osteoporosis outcomes.

To address this, an initial step is to longitudinally investigate frailty in a large population-based osteoporosis cohort of older women. To this end, using the Osteoporosis Risk Assessment study (OPRA) of women all aged 75 years at inclusion with reassessment at ages 80 and 85, the purpose of this initial study is to understand the distribution of frailty among older community-dwelling women and progression rate over 10 years, but also potential prediction of mortality and osteoporosis.

## Materials and methods

### Subjects

**Fig. 1:**
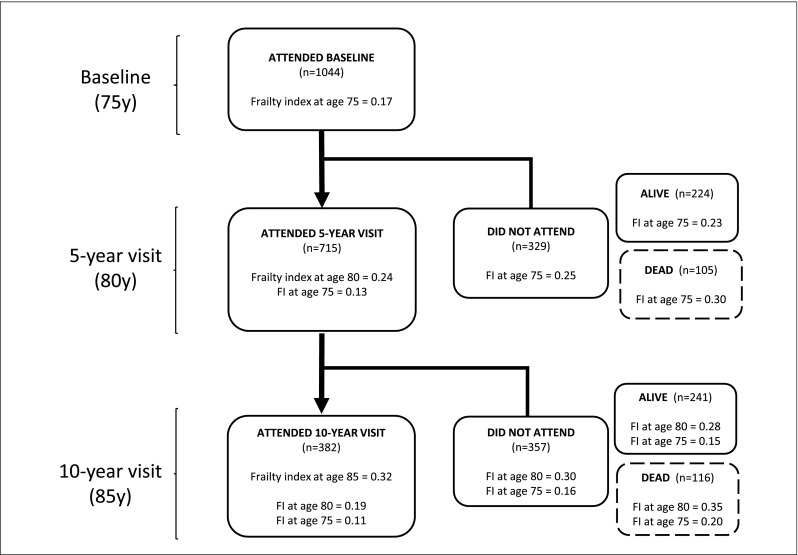
Frailty across participation at each visit; frailty index reported for attendees and non-attendees, dead or alive

In this study, we investigate 75-year-old community-dwelling women (the OPRA cohort) [16]. The cohort was randomly selected from population registries and women invited on their 75th birthday. No exclusion criteria were applied. A total of 1044 women participated in the baseline investigation between 1995 and 1999, representing a participation rate of 67%. Reasons for non-attendance have previously been detailed [17]. Follow-up investigations were performed at 5 years (age 80, *n* = 715 attended) and at 10 years (age 85, *n* = 382 attended). Similarly, reasons for non-attendance have been detailed [18] (Fig. 1).

The participants were extensively investigated at each visit. A questionnaire provided information on lifestyle (education, work, physical activity, smoking, and alcohol), health (medications, surgery, injuries, other diseases, food/nutrition, and hormonal function) falls, and fractures. The questionnaire was revised at follow-up to include supplemental information and events over the intervening 5 years. Self-estimated risk of falling was assessed using a Likert scale with 5 as the highest risk.

Physical assessment included balance (modified Romberg method), gait speed, and number of steps (30-m walk, 2 × 15 m with one turn) and thigh muscle strength (Biodex Medical Systems®, v4.5.0, Biodex Corporation, Shirley, N.Y., USA) as previously described [19]. Biochemical markers (CRP and creatinine) were assayed as described [18, 20]. BMD was measured using dual-energy x-ray absorptiometry (DXA) (GE Lunar, Madison, WI) [19] and the same machine used throughout. In this study, femoral neck BMD is used with osteoporosis being defined as a T-score below − 2.5. Precision of DXA was assessed by duplicate measurements on healthy individuals (precision error was 0.009–0.010 g/cm2 at FN). No drifts in phantom measured results were observed [21].

Mortality as date of death was acquired in October 2012, from the Swedish National Population Register (individuals still alive were a maximum 91.5 years of age).

Participants provided written informed consent, and the regional ethical review board at Lund University approved the study, which was performed according to the principles of the Helsinki Declaration.

### Study-specific frailty index

Being an osteoporosis cohort, we were unable to define frailty according to the most commonly used frailty phenotype [5]. Instead, using the principles of Searle et al. [22], we followed a stepwise process to construct an index with available data that allowed us to capture frailty across all assessment points.

As described in detail below, the final frailty index (FI) used in these analyses consists of 13 variables at all visits (Table 1). Covering a number of physiological domains, e.g., mobility, strength, co-ordination, and poly-medication, the index represents, for each OPRA participant, the number of “deficits in health.” The FI was calculated by dividing the number of deficits present by the total number of deficits examined, giving a score from 0.0–1.0, where a higher score indicates a higher frailty status. Where an individual had missing information for a particular variable, the total deficits were reduced by one.

**Table 1:** Components included in the OPRA-specific Frailty Index constructed at ages 75, 80, and 85

	OPRA-specific Frailty Index	Measurement units	Scoring or cut point
1	Daily physical activity	Categories 1–6 (1 = lowest; 6 highest)	Cat 1–3 = 1; cat 4 = 0.5; cat 5–6 = 0
2	Average time spent outdoors	Hours	< 1 h = 1; ≥ 1 h = 0
3	Gait—walking speed for 2 × 15 m	m/s	> 1.20 = 1; < 1.20 = 0
4	Gait—steps taken walking 2 × 15 m	No. of steps	< 54 = 0; > 54 = 1
5	Balance (2 legs, eyes closed)	Seconds	Failed test = 1; passed test = 0
6	Muscle strength—knee extension*	Nms	> 213 = 0; < 213 = 1
7	Diabetes	Yes/No	Yes = 1; No = 0
8	Cancer	Yes/No	Yes = 1; No = 0
9	Diseases affecting balance	Yes/No	Yes = 1; No = 0
10	Polypharmacy, using 5 or more medications	Yes/No	Yes = 1; No = 0
11	Self-estimated risk of falling	Categories 1–5 (1 = lowest; 5 highest)	Cat 1–5: 0.0; 0.25; 0.5; 0.75; 1.0
12	P-CRP	mg/L	> = 4.21 = 1; < 4.21 = 0
13	P-creatinine	umol/L	> = 82.02 = 1; < 82.02

*Voluntary maximal, isometric muscle strength of the right knee (knee extension at 90°) measured using a Biodex computerized dynamometer

To score variables (deficit present/non-present), we used either clinically relevant cut-points or identified the cut-off values by plotting the variable against an interim FI [22]. Categorical values were converted to binary values 1 (=deficit present) and 0 (=deficit absent); those with more than two categories were scored between 0 and 1 (e.g., high = 1.0; medium = 0.5; low = 0.0). To estimate cut-points of continuous data for dichotomization, curve estimation regression was performed, plotting potential frailty index variables against an intermediate frailty index. The resulting categories were then tested for differences in survival using Cox proportional hazard regression [22].

#### Frailty index development, construction, and validation

Searle et al. [22] recommend an index consisting of 30–40 variables. Since the availability of suitable data was limited at baseline, we constructed the index using the following approach.

Using data collected at the 5-year follow-up (age 80), a 40-variable index was first constructed, then to validate the method, prediction of mortality risk was tested using Cox regression (mortality risk HR 3.5 [95% CI, 2.5–4.8]). In the next step, the 40 variables were reduced to 10, considering availability at all time-points, and a 10-variable index was constructed (using data at age 80) and found equally predictive of mortality (HR 3.1 [2.4–3.9]). In an additional step, to ensure a wider coverage of biological domains essential for a measurement of frailty, additional variables (such as biomarkers) were added as covariates in logistic regression analysis to identify further variables associated with mortality risk. This resulted in the creation of a 15-variable index, which could be compared longitudinally across the complete duration of follow-up (full details available on request). For the purpose of the present analyses, the BMD variables was subsequently removed from the index, since it is the study outcome, as was BMI due to its strong correlation to BMD. Correlation between the 40- and 13-variable indices was high (Spearman’s *r*^2^ = 0.846). All indices were equivalently predictive of mortality.

### Statistical analyses

Descriptive statistics are reported as mean and standard deviation (SD), median, and IQR or frequency and percentage.

The frailty index, which shows a typically positively skewed (gamma) distribution [22], was used both as a continuous variable and stratified into quartiles (Q1 = lowest level of frailty; Q4 = highest level of frailty). Statistical comparisons were calculated overall or for Q1 vs Q4 as appropriate.

Annual linear progression of frailty over 10 years was calculated as the average, based on mean values of the whole cohort. For mortality, hazard ratios (HR) and 95% confidence intervals (95% CI) were estimated using Cox proportional hazard regression with the healthiest quartile (Q1) as the reference category. Time to death was 5 years, 10 years, or until end of study (i.e., October 2012). HRs are presented unadjusted.

For osteoporosis, differences in T-score between the frailty categories were estimated using the non-parametric Kruskal Wallis test.

Analyses were performed using SPSS version 22 (SPSS, Inc., Chicago, IL) and JMP SAS (SAS Institute, Cary, NC, USA). *p* values of < 0.05 were considered nominally significant.

## Results

Characteristics of the OPRA cohort, including frailty score components at ages 75, 80, and 85, are presented in Table 2. In general, the frailest women typically had poorer gait, balance and muscle strength, the highest CRP, more frequent polypharmacy, and the lowest albumin (a proxy for nutritional status) levels (data not shown).

**Table 2:** Key clinical characteristics of the OPRA cohort at age 75, 80, and 85

All variables at 75 years	Age 75 (baseline) *n* = 1044		Age 80 (5 year) *n* = 715		Age 85 (10 year) *n* = 382	
	Mean or No.	SD or %	Mean or No.	SD or %	Mean or No.	SD or %
Age (years)	75.2	(0.2)	80.2	(0.2)	85.2	(0.1)
Height (cm)	160.5	(5.7)	159.2	(5.8)	158.3	(5.8)
Weight (kg)	67.8	(11.7)	66.0	(11.6)	63.95	(10.9)
BMI (kg/m^2^)	26.3	(4.2)	26.1	(4.2)	25.5	(4.0)
OPRA-specific Frailty Score	0.17	(0.17)	0.24	(0.18)	0.32	(0.19)
Distribution of FI components						
Daily activity^1^	0.06	(0.19)	0.11	(0.23)	0.20	(0.26)
Average time spent outdoors (hours)	2.73	(1.33)	1.84	(0.87)	1.66	(0.78)
Gait—walking speed for 2 × 15 m (m/s)	1.31	(0.30)	1.20	(0.33)	1.10	(0.32)
Gait—steps taken walking 2 × 15 m	49.4	(9.8)	53.6	(11.7)	55.8	(12.3)
Balance (2 legs, eyes closed)(s)*	57.8	(10.6)	54.8	(14.6)	52.1	(17.5)
Balance (No. failing 60-s test)	47	(4.6%)	91	(12.7%)	75	(20.3%)
Muscle strength^2^ (nms)	267.9	(79.5)	247.3	(71.2)	218.3	(63.6)
Diabetes/cancer (%)	219	(21.0%)	178	(24.9%)	91	(24.1%)
Disease affecting balance (%)	201	(22.6%)	256	(35.8%)	184	(48.2%)
Self-estimated risk of falling (cat1–5)						
Low (1–2)	681	(75.4%)	491	(62.1%)	240	(63.8%)
Medium (3)	126	(14.0%)	129	(18.9%)	94	(25.0%)
High (4–5)	98	(10.6%)	61	(8.9%)	42	(11.2%)
Polypharmacy^3^ (%)	210	(20.1%)	175	(24.5%)	165	(43.2%)
P-CRP (mg/L)	3.9	(6.8)	3.7	(5.1)	3.4	(5.8)
P-creatinine (umol/L)	69.9	(0.60)	74.3	(19.9)	82.2	(1.20)

Mean (SD) unless otherwise stated. ^1^Daily activity calculated from the frailty threshold categories; ^2^voluntary maximal isometric muscle strength of the right knee (knee extension at 90°) measured using a Biodex computerized dynamometer; ^3^five or more medications; *not used in index

### Progression of frailty

**Fig. 2:**
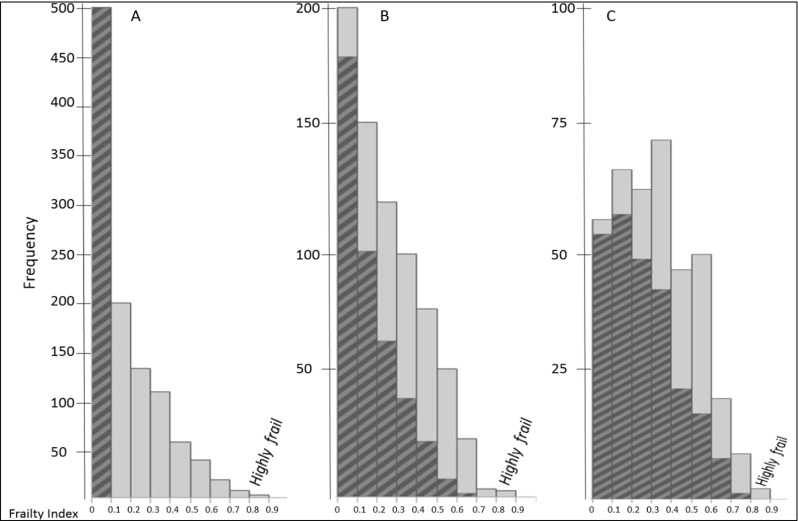
Frailty and change of frailty over time in older women assessed at baseline, 5-year and 10-year follow-up, tracking progression in those most robust at baseline (hatched area). The three histograms show the distribution of frailty index scores at each visit (baseline, 5 years, 10 years). The index is presented in decentiles (0.0–1.0). The hatched area in (**a**) represents the LEAST frail women at baseline, and their progression towards increasing frailty over the course of the study (panels **b** and **c**)

Over 10 years of follow-up, mean frailty increased giving an approximate annual linear frailty score progression of 6–7% (Table 2, Fig. 1). At baseline, the proportion scoring least frail (FI 0.0–0.1), i.e., most robust, constituted 48% of the cohort. At age 80 and 85, that proportion dropped to 25 and 14%, respectively. Among those rated least frail at age 75, although they progressed in frailty, the majority only reached intermediate levels (FI 0.2–0.6). As many as 11% had no change in frailty status and remained robust during the 10 years. Figure 2 illustrates the progression towards increased frailty among the participants.

### Mortality

Those who died during the first 5-year period had the highest average frailty scores at baseline (*n* = 105; mean FI 0.30, median 0.29); approximately similar to the mean FI at age 85. The same trend was observed tracing those who attended the 5-year visit and comparing their frailty score at the 10-year follow-up (Fig. 1).

**Fig. 3:**
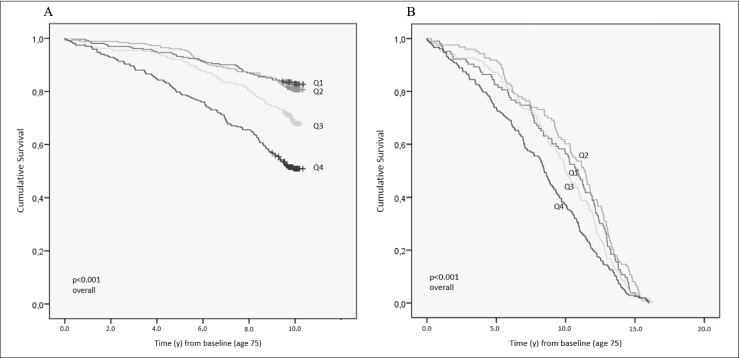
Mortality risk according to quartiles of frailty at age 75. **a** 10 years. **b** End of study

Mortality was substantially higher in the highest quartile of frailty compared to the lowest based on their baseline frailty score; after 10 years, 49.1% had died compared to 17.2% (*p* = 8.4 × 10^−15^). At 10 years, mortality was also higher in Q3 and 70% of those dead contained in Q3–Q4 (Table 3). The corresponding mortality risk over the first 5 years was highest in the frailest women (Q4 vs Q1; HR_unadj_ 3.26 [1.86–5.73]; *p* < 0.001) and continued to be elevated at 10 years (HR_unadj_ 3.58 [2.55–5.03]; *p* < 0.001) (Fig. 3). At age 85, only the least frail (i.e., most robust) had 2–3 times lower mortality, compared to the other quartiles.

**Table 3:** Frailty by quartiles at age 75, 80, and 85 and distribution of mortality and bone mineral density

	Low frailty (Q1)	Frailty (Q2)	Frailty (Q3)	Highly frail (Q4)	*p* value^#^ overall	*p* value Q1 vs Q4
All variables at 75 years (*n* = 1044)	*n* = 261	*n* = 254	*n* = 262	*n* = 267		
OPRA-specific Frailty Index (range)	0.00–0.02	0.03–0.12	0.13–0.27	0.28–0.88		
No. dead at 5 years (age 80 follow-up) (%)	18 (6.9)	11 (4.3)	21 (8.0)	55 (20.6)	5 × 10^−10^	1.1 × 10^−5^
No. dead at 10 years (age 85 follow-up) (%)	45 (17.2)	49 (19.3)	84 (32.1)	131 (49.1)	1 × 10^−5^	8.4 × 10^−15^
BMD—Femoral neck g/cm^3^	0.773 (0.131)	0.770 (0.136)	0.756 (0.136)	0.759 (0.150)	0.460	0.280
Bone density—femoral neck T-score	− 1.72 (1.09)	− 1.75 (1.13)	− 1.86 (1.14)	− 1.84 (1.25)	0.460	0.280
Osteoporosis—FN T-score < − 2.5 (*n*/%)	61 (24.6)	69 (28.3)	74 (30.3)	62 (29.4)	0.516	0.290
All variables at 80 years (*n* = 715)	*n* = 196	*n* = 158	*n* = 187	*n* = 174		
OPRA-specific Frailty Index (range)	0.00–0.10	0.11–0.22	0.23–0.38	0.39–0.85		
No. dead at 5 years (age 85 follow-up) *n* (%)	14 (7.1)	17 (10.8)	32 (17.1)	53 (30.5)	< 0.001	9.8 × 10^−9^
No. dead at end of study (%)	64 (32.7)	55 (34.8)	97 (51.9)	115 (66.1)	2 × 10^−11^	1 × 10^−10^
BMD—femoral neck g/cm^3^	0.720 (0.114)	0.713 (0.123)	0.714 (0.126)	0.702 (0.153)	0.652	0.221
Bone density—femoral neck T-score	− 2.17 (0.95)	− 2.23 (1.03)	− 2.22 (1.05)	− 2.31 (1.27)	0.652	0.221
Osteoporosis—FN T-score < − 2.5 (*n*/%)	74 (38.3)	68 (44.2)	81 (45.8)	75 (47.5)	0.323	0.103
All variables at 85 years (*n* = 382)	*n* = 102	*n* = 95	*n* = 100	*n* = 85		
OPRA-specific Frailty Index (range)	0.00–0.17	0.18–0.31	0.32–0.46	0.47–0.83		
No. dead at end of study (%)	14 (13.7)	27 (28.4)	27 (27.0)	37 (43.5)	1.2 × 10^−4^	6 × 10^−6^
BMD—femoral neck g/cm^3^	0.699 (0.128)	0.700 (0.145)	0.662 (0.125)	0.699 (0.148)	0.154	0.974
Bone density—femoral neck T-score	− 2.34 (1.06)	− 2.34 (1.21)	− 2.65 (1.04)	− 2.34 (1.23)	0.154	0.974
Osteoporosis—FN T-score < − 2.5 (*n*/%)	50 (49.5)	42 (45.7)	53 (55.8)	35 (44.3)	0.412	0.548

Reported values are means, unless otherwise stated. ^#^*p* values calculated by ANOVA, *t* test, Fisher’s exact test, or Chi-square as appropriate

### Participation

Study participation may serve as an indicator of societal participation. Women who were alive but did *not* attend 5-year follow-up at age 80 were more frail at baseline than those who attended again (mean FI 0.23, median 0.19 vs FI 0.13, median 0.09). Further demonstrating the applicability of this frailty index in a long-term perspective, initial baseline frailty score was lowest in those who attended 10-year follow-up (FI 0.11, median 0.08), and increased stepwise in those who were alive but did not attend (FI 0.15, median 0.10) and those who had died (FI 0.20, median 0.18) (Fig. 1).

### Osteoporosis and frailty

Aging is associated with osteoporosis and since this cohort was specifically designed for this purpose, we tested the association between frailty and osteoporosis. The proportion with osteoporosis increased with age as expected in the population overall; at baseline, 28.1% were osteoporotic rising to 49.0% after 10 years. At age 75, femoral neck BMD was 0.773 g/cm^2^ (SD 0.131) in the least frail compared to 0.759 (SD 0.150) in the frailest, and not statistically different. After adjustment for BMI, BMD was significantly associated with frailty (overall; *p* = 0.0006 and Q1 vs Q4 *p* = 0.0003). The pattern was similar at age 80, while femoral neck BMD at age 85 was similar across frailty quartiles (Table 3), adjustment for BMI did not result in a statistically significant difference (data not shown).

## Discussion

In this study, we show how frailty is distributed in a population-based cohort of older community-dwelling women where the majority are still in relatively good health at age 75. We also show the progression of frailty with advancing age, noteworthy being the pattern of change among those initially least frail, while the higher mortality among the most frail is as expected. Our findings highlight the possibility of, and also the value of, estimating overall health in older people by objectively evaluating frailty as part of prognosticating healthy aging and future adverse events.

How to best measure frailty is widely discussed and many instruments have been suggested [4, 23]. Our frailty index was developed according to the fairly simple philosophy of “The more individuals have wrong with them, the higher the likelihood that they will be frail” suggested by Rockwood and Mitnitski; meaning that these “wrongs” or deficits will mirror impaired and aging-associated processes at a cellular level, and that more deficits within different physiological systems are reflecting the generalized syndrome considered essential for frailty [6, 22, 24]. Our cohort was designed to assess osteoporosis risk in older women and not for estimating frailty; however, we show that is possible to use the variables available to construct an informative frailty index highly predictive of mortality. In accordance with the stated principles, the method allows for a varying number and types of variables to be used as long as they follow the basic rules [22].

Approximately half of the women were in the least frail category (FI 0.0–0.1) (i.e., were most robust) at age 75. Five years later, this was halved and at age 85 halved again as deficits accumulate. Frailty increased by 6 to 7% per year, which is higher than in some studies, most likely because we are assessing same-aged individuals as they age while other studies compare the difference by chronological year [22, 24–26]. Furthermore, recognizing frailty as a state where reserve capabilities are reduced, it is reasonable to assume that, once a threshold has been passed, frailty evolves at a faster pace. Such a threshold has not yet been defined, but our data indicate a clinical cut-off of approximately 0.27. Given our data describing the pattern of progression over many years in older women and given that frailty is considered dynamic and hence potentially reversible, our findings highlight the need to observe frailty status together with advancing age to ensure timely interventions. Currently, the evidence supporting interventions to reverse or minimize the rate of decline are varied but most rely on nutrition and training [27–31].

Mortality was highest in the most frail; at age 75 and during the following 5 years half of all those dead were among the frailest and the risk of dying more than three times that of the least frail. But those in the next quartile (Q3) also had a higher mortality over 10 years, suggesting their pre-frailty status. The same pattern was apparent when frailty was assessed at age 80. In contrast, and mirroring the age-related shift towards increased frailty, at age 85, all but the most robust (i.e., least frail) had a 2–3 times higher mortality. One interpretation of this is that it is most useful to identify signs of frailty at earlier ages to allow for appropriate intervention. To put this into perspective, those who died within 5 years of baseline (between age 75 and 80) had a mean FI equivalent to someone 10 years older, i.e., comparable to those attending at age 85, meaning they were 10 years more frail. Those who did not attend the 5-year follow-up had a baseline FI similar to those attended at age 80, suggesting they were 5 years more frail.

The osteoporotic patient is assumed to be more frail. Therefore, we hypothesized that the frailest women would have lower bone density and a higher proportion with osteoporosis. This was the case, but after adjustment for BMI. Frailty in relation to bone density is only addressed in a few studies and with inconsistent results as a consequence of small sample, diverse populations, and frailty definitions [32, 33], yet frailty is very relevant to osteoporosis since its clinical outcome of fracture encompasses a wider spectrum than BMD alone (which we are addressing in another study). Furthermore, an additional observation among these community-dwelling women is that BMI was higher in those with higher frailty, indicative of an accumulation of conditions resulting in an overall decreased health status and reduced activity. This also suggests that assessment of bone should not be overlooked in women with higher body weight, but overall poor health status.

### Limitations and strengths

Firstly, one potential limitation is that our frailty index was derived and applied in the same population and external validation of the index has not been performed. While validation would be valuable, this is however, part of the problem in the emerging field of frailty and mirrors the lack of consensus and inconsistency across studies in terms of collected information. Further to this, making direct comparison with other studies is difficult; however, in our index, the cut-off for frailty coincides with the lower limit of Q4 and while a consensus threshold is lacking; this is close to the empirical cut-off point of > 0.25 for a frailty index based on accumulated deficits as described by Rockwood [6].

Secondly, our index has fewer variables than the suggestion of 30–40; however, in its development, we demonstrate a very high correlation and an almost identical ability to predict mortality between a 40-item index and the 13-item index used in this study. This most likely reflects the high inter-relationship between the included variables, whereby one variable can capture and substitute multiple variables. It can also be argued that this high redundancy between variables is an advantage as it indicates the possibility to use simpler constructs and facilitate use. Thirdly, due to constraints from the original study design and subsequent lack of information in certain domains, data on social and mental factors are unfortunately not included. Fourth, being a longitudinal study of older persons, there is an inherent problem of loss to follow-up and a potential bias of healthy participants. Indeed, we also show that those continuing in the study are less frail, and with regard to mortality, this is not problematic, but a loss of power may occur for other outcomes, although the descriptive information is still robust. Fifth, caution should be exercised in terms of generalizing the findings to other populations such as younger women or other ethnicities.

Strengths of this study include that the participants are 75-year-old community-dwelling rather than institutionalized women, representing a pivotal period for continued healthy aging or deteriorating health. The fact that all women were at the same age at inclusion is advantageous as it minimizes the influence of chronological age on accumulated health deficits. Another strength is the provision of longitudinal data for up to 15 years allowing us to quantitatively assess change in frailty status with advancing age. Additionally, we demonstrate that it is possible to develop a meaningful frailty index from available data and with the same discriminatory ability as a more comprehensive, larger item index. This is important since research on frailty in relation to osteoporosis is still in its infancy but potentially beneficial for future research. Taken together, this study contributes with data on frailty in averagely healthy older women including tracking over time and its association to bone health.

In conclusion, the relevance of this study lies in demonstrating the pattern of frailty longitudinally in older community-dwelling women and its association to mortality up to 15 years. Frailty was associated with a threefold increased risk of death in both a short and longer perspective with a higher frailty score being equivalent to being chronologically five to 10 years older. Conversely, only one in ten older women escaped progression of frailty. In addition, higher frailty is associated with osteoporosis, despite the fact that the frailest individuals may have a higher BMI.
